# *Zinc Finger Protein8* (*GhZFP8*) Regulates the Initiation of Trichomes in *Arabidopsis* and the Development of Fiber in Cotton

**DOI:** 10.3390/plants13040492

**Published:** 2024-02-08

**Authors:** Yongchang Liu, Xiaomei Ma, Ying Li, Xiaoyu Yang, Wenhan Cheng

**Affiliations:** 1 College of Bioengineering, Jingchu University of Technology, Jingmen 448000, China; liying19830923@126.com (Y.L.); 13053549857@163.com (X.Y.);; 2 Cotton Research Institute, Xinjiang Science Academy of Agriculture and Reclaimation, Shihezi 832000, China; maxm_09@163.com

**Keywords:** *Gossypium hirsutum*, C_2_H_2_, zinc finger protein, trichome, cotton fiber

## Abstract

Cotton is one of the most important natural fibers used in the textile industry worldwide. It is important to identify the key factors involved in cotton fiber development. In this study, *zinc finger protein8* (*GhZFP8*) encoding a C_2_H_2_ transcription factor (TF) was cloned from cotton. qPCR showed that the transcripts of *GhZFP8* in cotton were detected in the leaves and fibers at 3, 6, and 30 days post-anthesis (DPA), but not in the roots, stems, or flowers. The overexpression of *GhZFP8* increased the trichome number on the siliques, leaves, and inflorescence, but inhibited the growth. The expression of trichome development and cell-elongation-related genes decreased obviously in *GhZFP8* overexpressor *Arabidopsis*. Indole-3-acetic acid (IAA) and 1-Aminocyclopropanecarboxylic acid (ACC) contents were much higher in *GhZFP8* overexpressors than that found in the wild type, but the gibberellin (GA) content was lower. The interference of *GhZFP8* in cotton caused smaller bolls and shorter fibers than that of the control. The results of DNA affinity purification (DAP)-seq showed that GhZFP8 could bind to the promoter, exon, intron, and intergenic region of the target genes, which are involved in photosynthesis, signal transduction, synthesis of biomass, etc. Our findings implied that GhZFP8 processed multiple biological functions and regulated the development of cotton fiber.

## 1. Introduction

The trichome of *Arabidopsis* is a typical single-cell structure derived from epidermal cells by cell fate determination, cell specialization, and morphogenesis. The epidermal growth factors expressed in the epidermal cells can activate the expression of the trichome suppressor. The expression of inhibitors determines whether the cells eventually develop into trichomes [1]. In this pathway, there are two kind of regulators, including positive regulators, such as GL1, WER, MYB23, GL3, EGL3, and TTG1, and negative regulators, such as CPC, TRY, ETC1, and TCL1. GL1 and TTG1 can interact with WD40 protein GL3/EGL3 to form a BMW complex. This can activate the expression of *GL2* and *TTG2* and finally promote the differentiation of epidermal cells into trichomes [2]. However, the positive regulators activated by the BMW complex, such as GL2, TTG2, SIM, and RBR1, can inhibit the expression of some genes related to cell division, such as *CYCD3;1* and *CYCB1;2*, making the cell stop division, enter internal replication, and eventually differentiate into trichomes [3]. Negative regulators can shuttle between cells and compete with GL1 to bind GL3/EGL3, therefore, the complex cannot promote the expression of downstream activators, which results in the failure of epidermal cells to differentiate into trichomes [2].

ZFPs are the largest group of TFs involved in various biological processes, including pollen development [4], shoot development [5], root hair initiation and elongation [6], and the response to abiotic stresses [7], through transcriptional activation or inhibition, binding to RNA, and protein interaction. The conserved cysteine and histidine can combine with a zinc ion to form a finger-like structure and then bind to DNA to inhibit or activate gene expression. Recent studies have shown that C_2_H_2_-ZFPs, such as GIS, GIS2, GIS3, and ZFP5/6/8, regulate the development of trichome in *Arabidopsis* by acting on the BMW complex. ZFP5 can directly regulate the expression of *GIS*, *GIS2*, and *ZFP8*, and then GIS can increase the expression of *GL1* and *GL3* [8]. The trichome density of *ZFP8* and *GIS2* RNAi plants was significantly reduced in the flower organs, stems, and leaves [9,10]. GIS3 regulated the initiation of trichomes in *Arabidopsis* and acted upstream of *GIS*, *GIS2,* and *ZFP8* [11]. The trichomes of *Arabidopsis* and cotton fiber are derived from epidermal cells and are non-glandular single cells. In cotton, *GhSIZ1*, homologous of *AtZFP5*, was induced by NaCl, PEG6000, and cold treatment, and expressed primarily in the fiber of the early and advanced stage of fiber development [12]. Several *GhZFP8* subfamily members have a high expression level of fiber and are regulated by phytohormone [13]. In another paper, *GhZFP8* expression showed a high transcription level in the ovules at 0- and 1DPA [14]. Our previous study showed that *GhZFP8*, encoding a protein sharing 62% amino acid identifying with AtZFP8, was induced by abiotic stress and regulated the response of transgenic *Arabidopsis* to high salinity and abscisic acid (ABA) [15]. All studies have indicated that ZFPs might localize to the key point of cotton fiber development, especially GhZFP8 and its homologous genes.

In this study, we analyzed the expression pattern of *GhZFP8* during the whole process of fiber development and in different cultivars. We also explored its biological function on the trichome initiation in *Arabidopsis* and the fiber development in cotton. DAP-seq was performed to identify the target genes of GhZFP8. All of the findings suggested that GhZFP8 plays a vital role in the development of cotton through complex pathways.

## 2. Results

### 2.1. The Expression Pattern of GhZFP8 in Different Organs

**Figure 1:**
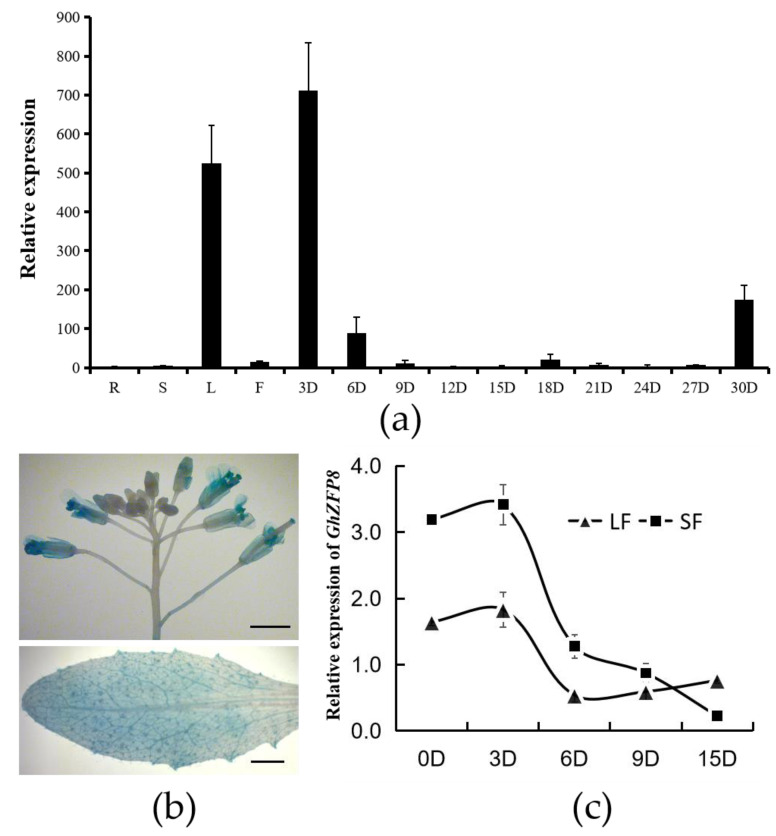
Expression pattern of *GhZFP8* in different tissue and varieties. (**a**) Expression pattern of *GhZFP8* in different tissues. R: Root; S: Stem; L: Leaf; F: Flower; 3 D, 6 D, 9 D, 12 D, 18 D, 21 D, 24 D, 27 D, and 30 D stand for cotton fiber of 3, 6, 9, 12, 18, 21, 24, 27, and 30 DPA, respectively. (**b**) The expression of GUS driven by the *GhZFP8* promoter in overexpressor *Arabidopsis*, flower and leaf, Bar = 0.25 cm. (**c**) The expression pattern of *GhZFP8* in the fibers of two cultivars. Here, 0 D, 3 D, 6 D, 9 D, and 15 D stand for cotton fiber of 0, 3, 6, 9, and 15 DPA, respectively. Data represent the mean ± SD of three replicates.

To check the expression pattern of *GhZFP8*, the total RNA was extracted from different tissues of cotton. qPCR was performed to check the transitional level of *GhZFP8*. Transcripts of *GhZFP8* were detected in the leaves and fibers of 3, 6, and 30 DPA, with the highest level being found in the fibers of 3 DPA (Figure 1a). GUS staining showed that the expression of GUS could be driven by the *GhZFP8* promoter in the flowers and leaves of *Arabidopsis*, especially at the bottom of trichomes (Figure 1b). To investigate the expression of *GhZFP8* in different varieties, two cultivars possessing different fiber lengths were selected (Table S1). The expression level of *GhZFP8* was much higher in the variety with long fibers (LF) than that found in the variety with short fibers (SF), and also reached the highest level at 3 DPA, then reduced obviously (Figure 1c).

### 2.2. Correlation of GhZFP8 to the Parameters of Cotton Fiber

**Figure 2:**
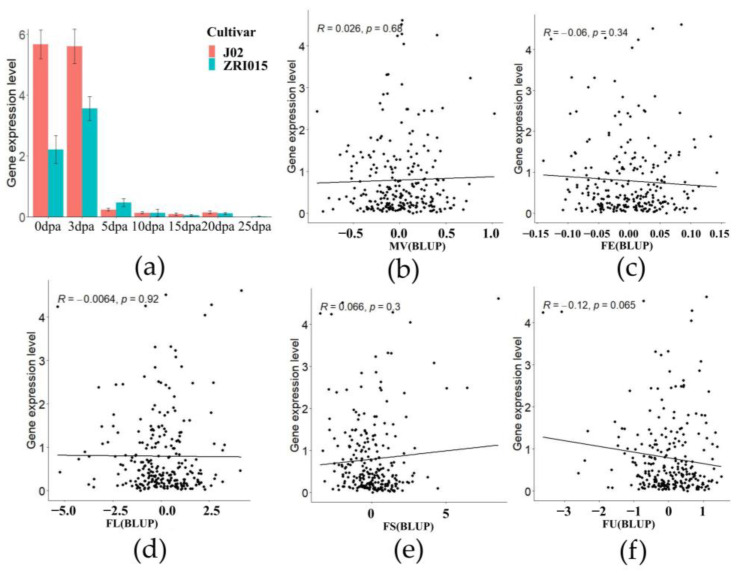
Correlation between *GhZFP8* expression and different parameters of cotton fiber. (**a**) The *GhZFP8* expression in the cotton fiber of J02 and ZRI015. Data represent the mean ± SD of three replicates. (**b**–**f**) Correlation of *GhZFP8* with different parameters of cotton fiber. MV (**b**), FE (**c**), FL (**d**), FS (**e**), and FU (**f**) are abbreviations for the fiber’s micronaire value, fiber elongation, fiber strength, and fiber uniformity, respectively.

In J02 and ZRI015, *GhZFP8* was highly expressed in the fibers of 0 and 3 DPA, but was low in the fibers of the other time points, which was consistent with the results shown in Figure 1a,c. The expression of *GhZFP8* was much higher in J02, which had longer fibers than ZRI015 (Figure 2a). In order to explore the relationship between *GhZFP8* and the development of cotton fiber, the correlation between the *GhZFP8* expression and the different parameters of cotton fiber were analyzed. The results showed that there was no significant correlation between *GhZFP8* and fiber elongation (FE), micronaire value (MV), fiber length (FL), and fiber strength (FS) (Figure 2b–e). Apart from those four cotton fiber parameters, the correlation between *GhZFP8* and FU was most significant, but its correlation coefficient was only −0.12, and the *p*-value was 0.065 (Figure 2f). *GhZFP8* had a high transcriptional level at 0 and 3 DPA, but the cotton fiber used for the correlation analysis was harvested on 15 DPA, when *GhZFP8*’s expression was very low.

### 2.3. Subcellular Localization of GhZFP8 in Tobacco Leaves

**Figure 3:**
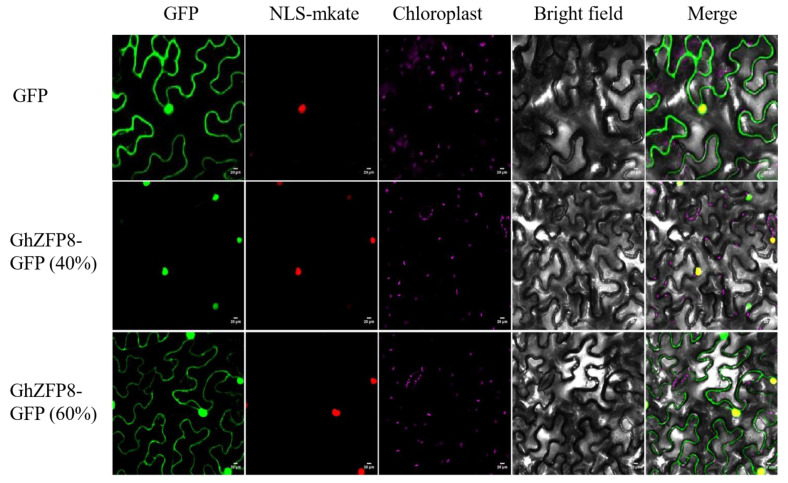
Subcellular localization of GhZFP8 in *Nicotiana benthamiana* leaves. Confocal section of tobacco leaves expressing GFP (**upper** panels) and GhZFP8-GFP (**middle** and **bottom** panels), respectively, Bar = 20 µm. The green fluorescence was the fluorescence of GFP and GhZFP-GFP; The red fluorescence was the fluorescence of NLS-mkate; The pink fluorescence was the autofluorescence of chloroplast; The yellow fluorescence was the fluorescence of GFP and RED merge.

In order to check the localization, the coding sequence of *GhZFP8* was fused with GFP. *GhZFP8*-*GFP*-fused genes were expressed and driven by a CaMV35S promoter in tobacco leaves. The results showed that GFP was located in the nuclei and cytoplasm (Figure 3 upper panels), while the fluorescence of GhZFP8-GFP in about 60% of the cells was distributed in the cytoplasm and nucleus (Figure 3 middle panels). In addition, GhZFP8-GFP was also detected in the nucleus in only about 40% of the cells (Figure 3 bottom panels), which could merge with NLS-mKate perfectly. This demonstrated that GhZFP8 was a nuclear-localized and cytoplasm-localized protein.

### 2.4. Overexpression of GhZFP8 Enhanced Formation of Trichomes

**Figure 4:**
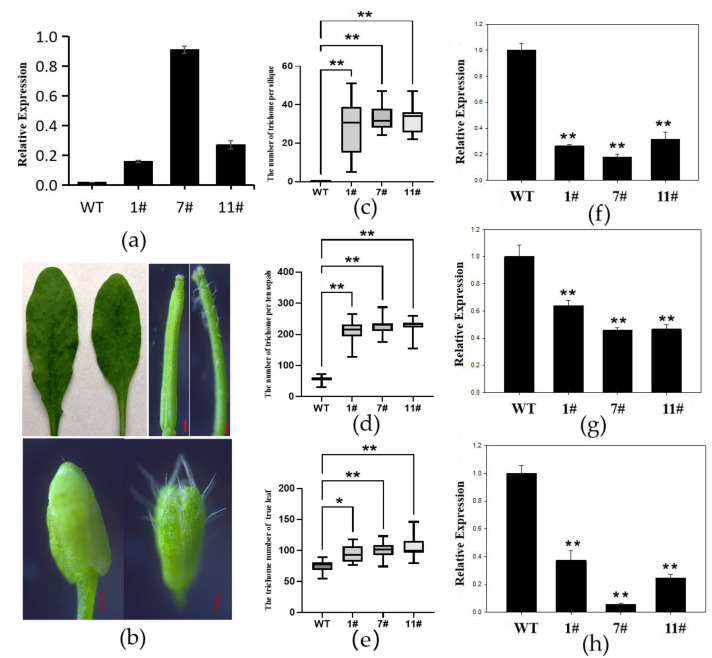
Trichome and gene analysis of *GhZFP8* overexpressor *Arabidopsis.* (**a**) The relative expression of *GhZFP8* in overexpressors. (**b**) Rosette leaf, silique, and bud of WT (**left**) and *GhZFP8* transgenic *Arabidopsis* (**right**), Bar = 1 mm. (**c**–**e**) The statistical analysis of trichomes on silique (**c**), sepal (**d**), rosette leaf (**e**). (**f**–**h**) Expression of *TRY1* (**f**), *ETC1* (**g**) and *TCL1* (**h**) in *GhZFP8* overexpressor *Arabidopsis*. RNA was isolated from the leaves of 21-day-old plants, and qRT-PCR was performed to check the expression of genes involved in trichome development. The expression of *ACTIN2* was used as a reference gene. Data represent the mean ± SD of three replicates. Significant differences compared with WT (one-way ANOVA multiple comparisons): * *p* < 0.05; ** *p* < 0.01.

To explore the biological function, an overexpressor *Arabidopsis* of *GhZFP8* driven by a 35S promoter was generated. About 45% of the overexpressors had much more trichomes, but all lines had a smaller plant size than the WT. qPCR was performed to check the transcripts of *GhZFP8*. The result showed that the transcripts were almost undetectable in the WT, and the relative expression of *GhZFP8* in the overexpressor plants was much higher (Figure 4a). The number of trichomes on the flowers and leaves increased, and ectopic trichomes were visible on the silique surfaces (Figure 4b). The WT plants had no trichomes on their silique surfaces, while 25–38 trichomes were observed on the silique of the *GhZFP8* overexpressors (Figure 4c). There were 2–3 trichomes on the WT flowers, but more than 20 on the *GhZFP8* overexpressors (Figure 4d). Compared to the rosette leaves of the WT (76), the trichome numbers were 92, 99, and 103 for the different overexpressors, respectively (Figure 4e).

In order to explore the molecular mechanisms of the *GhZFP8* function in trichome formation, qPCR was performed to check the expression of trichome-development-related genes using a gene-specific primer (Table S2). The results showed that the expression of *TRY1-*, *ETC1-*, and *TCL1*-encoded negative regulators was much lower than that found for the WT (Figure 4f–h). The genes encoding a positive regulator, such as *GL1*, *GL2*, *GL3*, *EGL3*, *TTG1*, etc., were also downregulated in the transgenic lines, except for *WER1* (Figure S1). Our results showed that *GhZFP8* promoted the initiation of trichomes by inhibiting the expression of negative regulators.

### 2.5. Overexpression of GhZFP8 Inhibited the Growth of Overexpressor Arabidopsis

**Figure 5:**
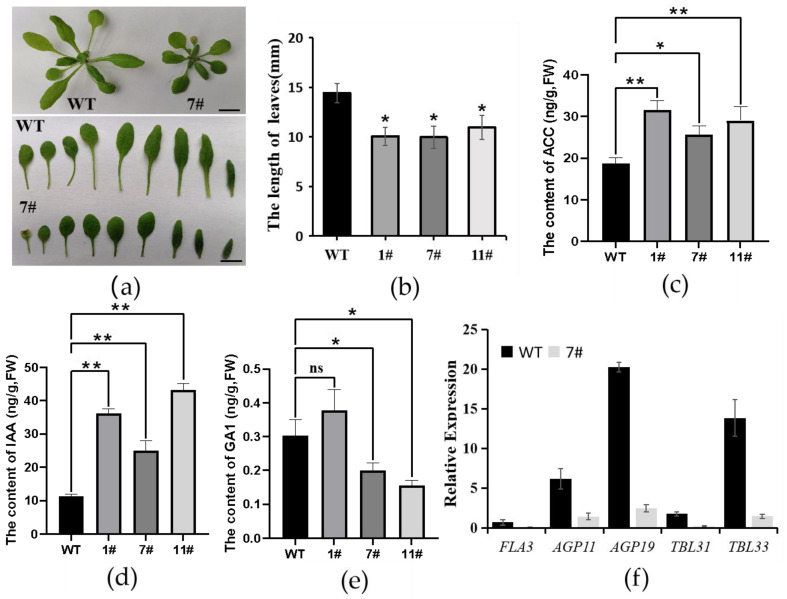
Overexpression of *GhZFP8* inhibited the growth of overexpressor *Arabidopsis.* (**a**) Phenotype of the overexpressor plant of *GhZFP8*, Bar = 1 cm. (**b**) Statistical analysis of the length of the fifth leaf. (**c**–**e**) The content of ACC (**c**), IAA (**d**), and GA (**e**) in the *GhZFP8* overexpressor and WT. (**f**) The expression of genes related to cell extension in the transgenic line and WT. Significant differences compared with WT (one-way ANOVA multiple comparisons): * *p* < 0.05; ** *p* < 0.01; ns, not significant.

The overexpression of *GhZFP8* also inhibited the growth of overexpressor *Arabidopsis*. The leaves of the *GhZFP8* overexpressors were much shorter than those of WT, which made the overexpressor plants look smaller (Figure 5a,b). To explore the change in plant size, the contents of the phytohormones were measured. The contents of ACC and IAA in all three lines were higher than those in the WT, while the content of GA was lower (Figure 5c–e). qPCR was performed to check the expression of genes related to cell extension. The results showed that the expression of *FLA3*, *AGP11*, *AGP19*, *TBL31,* and *TBL33*, which were positively correlated with cell extension, decreased dramatically (Figure 5f). Therefore, we speculated that *GhZFP8* might negatively regulate the growth by inhibiting gene expression and changing the hormone content.

### 2.6. Interference of GhZFP8 Regulated the Growth of Cotton

**Figure 6:**
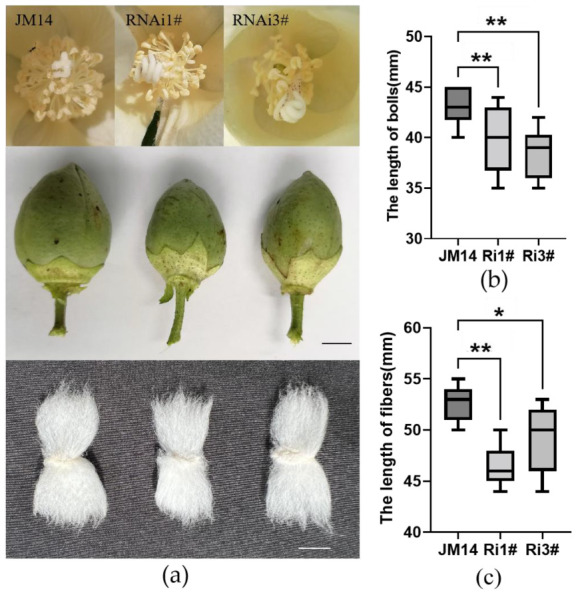
Phenotype of *GhZFP8* RNAi transgenic cotton and JM14. (**a**) Phenotype of flowers, bolls, and fibers of *GhZFP8* and JM14, Bar = 1 cm. (**b**) Statistical analysis of the length of bolls. (**c**) Statistical analysis of the length of fibers, *n* ≥ 12. Significant differences compared with WT (one-way ANOVA multiple comparisons): * *p* < 0.05; ** *p* < 0.01.

To explore the function of *GhZFP8* in the development of cotton, the expression of *GhZFP8* was interfered in upland cotton cultivar JM14, and four *GhZFP8* RNAi transgenic plants were generated. qPCR was performed to detect the transcripts of *GhZFP8* in RNAi transgenic lines, and the result showed that the transcription was inhibited significantly in all lines, especially in Ri2# and Ri4# (Figure S2). The interference of *GhZFP8* caused bad fertility, because the RNAi transgenic lines generated less pollen than JM14, especially in Ri2# and Ri4#, which had almost no pollen (Figure 6a). Meanwhile, Ri1# and Ri3# had smaller bolls and shorter fibers than JM14 (Figure 6a,b).

### 2.7. Identiffcation of GhZFP8-Binding Region by DAP-seq

**Figure 7:**
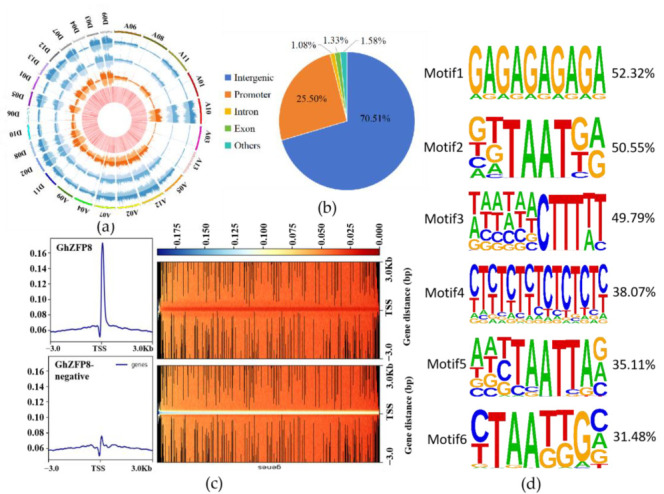
DAP-seq analysis of cotton GhZFP8. (**a**) The distribution of reads on the cotton genome. In order, from outside to inside, the outermost circle is the genome length scale, measured in kilobytes. The other circles represent the distribution histograms of reads on the positive and negative strands of the genome in samples of GhZFP8-negative (Orange) and GhZFP8-DAP (Blue), with the innermost red showing the gene distribution. (**b**) Statistics of distribution regions of peaks on gene functional elements. The promoter represents the region of 0–3 Kb upstream of the transcription start site (TSS). “Others” represents the locations of binding sites in 3′UTR (0.01%) and 5′UTR (0.34%), 300 bp downstream of TSS (1.23%). (**c**) Distribution map of peaks in the 3-kb region upstream and downstream of TSS. The horizontal axis represents position information, while the vertical axis represents the standardized signal value (CPM value). (**d**) Conservative motif found in the putative target genes of GhZFP8.

It is known that TF can bind to the promoter or other regions of downstream genes, which is helpful to the activation or suppression of their expression. To find out the putative target genes of GhZFP8, DAP-seq was performed using genomics DNA of Jimian14- and GhZFP8-Halo-fused proteins. The results of the sequence showed that 8761 binding regions of GhZFP8 were detected in the whole genome of cotton, but the number of binding peaks was different on each chromosome of cotton (Figure 7a). Compared to the negative control, the distribution of binding peaks was abundant near the transcription start site (TSS), which accounted for 25.50% of the total peaks, while 2.31% of the total peaks were distributed in the intron and exon (Figure 7b,c). To identify the binding motifs of GhZFP8 in cotton, prediction was performed in HOMER (http://homer.ucsd.edu/homer/download.html, accessed on 23 September 2023). The result showed that 117 known motifs were found in cotton, in which motif A and motif B existed in more than 50% of total target genes, while 38 de novo motifs were found and existed in less than 3% of the total target genes (Figure 7d, Table S3).

### 2.8. GhZFP8 Binds to Genes Associated with Cotton Fiber Development

**Figure 8:**
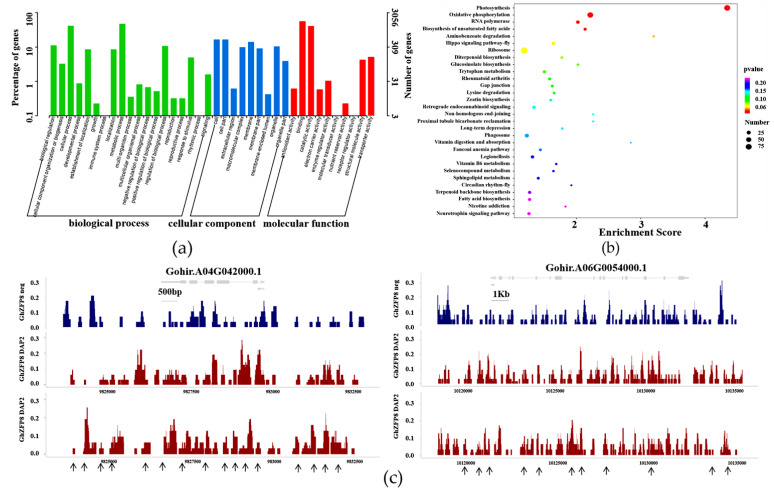
Target genes analysis of GhZFP8 in cotton. (**a**) GO analysis of GhZFP8 putative target genes. (**b**) KEGG analysis of GhZFP8 putative target genes. (**c**) Binding sites in two putative target genes. The horizontal axis represents the position of peaks, and the vertical axis represents standardized signal values (RPGC values). The gray box represents the exon of the gene, and the line represents the intron. The blue color in subfigure represent binding peaks in negative sample, and the red color represent binding peaks in two DAP samples. Arrows represent the predicted binding sites of GhZFP8.

Go and KEGG analyses were employed to explore the mechanism of GhZFP8. The results indicated that the binding-peak-related genes were related in terms of catalytic activity, binding, cell components, metabolic processes, etc., (Figure 8a). Many of the genes were known as important components of photosynthesis, including Gohir. A04G042000 (uroporphyrinogen decarboxylase), Gohir. A06G122200 (FAD-linked oxidase), Gohir. A13G075900 (FAD/NAD-binding protein), Gohir. A04G053130 (NADH ubiquinone oxidoreductase), Gohir. D08G108108 (NADH quinone oxidoreductase), and Gohir. A10G124100 (chlorophyllase hydrolase). Some genes were involved in signal transduction, including Gohir. A01G138600 (calmodulin-binding protein), Gohir. D03G071400 (calcineurin-like phosphoesterase), Gohir. A03G076800 (calcium-ion-binding protein), Gohir. A06G054000 (GTPase-activating protein GYP1), and Gohir. A10G135900 (small GTP-binding protein. GhZFP8 could bind to some genes related to the synthesis of biomass, including Gohir. D04G151200.1 (cellulose-synthase-like protein), Gohir. D11G223300 (acyl-CoA oxidase), Gohir. A11G232000 (very-long-chain enoyl-CoA reductase), Gohir. A11G200216 (trehalose-phosphatase), etc., (Figure 8b,c; Tables S4–S6).

## 3. Discussion

### 3.1. GhZFP8 Might Function as a Suppressor in Arabiodosis

The molecular mechanism for regulating the development of trichomes is relatively conservative in plants. In recent years, several homologous genes regulating the formation of trichomes in *Arabidopsis* were shown to affect the development of trichomes in other plant species [16,17,18]. In this study, the overexpression of *GhZFP8* in *Arabidopsis* increased the number of trichomes in the leaves, flowers, and siliques, similar to the function of *AtZFP8*, *AtGIS*, and *AtGIS2*. As a positive regulator, AtGIS could activate the expression of *GL1*; however, the targets of AtZFP8 and AtGIS2 have not been identified [9]. A previous study showed that *GhZFP8* encodes a C_2_H_2_ zinc finger protein containing a DNA-binding motif (QALGGH) and a suppression motif (DLNbox/EAR) in its C-terminus [15]. In *GhZFP8* overexpressor plants, not only many positive regulators, including *GL1*, *GL2*, *GL3*, *GIS*, *GIS3*, *GA1*, *TTG1*, and *EGL3*, but also some negative key factors, such as *TCL1*, *ETC1*, and *TRY*, were downregulated. In *Arabidopsis*, the suppression of *TCL1*, *ETC1*, and *TRY* could increase the number of trichomes [19,20]. The increased numbers of trichomes in *GhZFP8* overexpressor *Arabidopsis* might result from the inhibition of some negative regulators.

Previous studies have shown that disordering the auxin and ethylene content or the signaling pathway could inhibit the growth and development of *Arabidopsis* [21,22]. In fact, AtZFP8, AtGIS, JcZFP8, and NbGIS play important roles in regulating trichome formation through the hormone signaling pathway [9,17,18,23]. A previous study indicated that the GhZFP8 subfamily has high expression levels during the early stage of fiber development and were regulated by BR and GA treatment [13]. In *GhZFP8* overexpressors, the contents of IAA and ACC were much higher than those found in the WT, but the GA content was lower, which may account for the small plant size of *GhZFP8* overexpressor *Arabidopsis*. Compared to the WT, many genes involved in cell wall biogenesis were downregulated, such as *FLA3*, *AGP11*, *AGP19*, *MYB103*, *AGP8*, *AGP9*, *FLA3*, *TBL31*, and *TBL33*. Arabinogalactan proteins are important components of a cell wall and are key factors for cell elongation and reproductive processes. Abnormal expression of AGP genes could inhibit pollen tube growth, pollen development, female gametogenesis, and stem elongation [24,25,26,27]. The interference of *FLA3* encoding fasciclin-like arabinogalactan protein caused abnormal grains, but its overexpression inhibited the elongation of the stamen filament, reduced female fertility, and led to short siliques in *Arabidopsis* [28]. Xylan is a heterogeneous polysaccharide in plant cell walls and is the main component of plant hemicellulose. *TBL3*, *TBL31*, *TBL32*, and *TBL33* are homologous to *ESK1* and have a redundant function in catalyzing 2-O- and 3-O-monoacetylation of xylan. A mutation of *TBL3/31* and *ESK1,* or *TBL32/33* and *ESK1,* could inhibit the growth of *Arabidopsis* [29,30]. In a word, the overexpression of *GhZFP8* inhibited growth by disordering the content of the phytohormone and gene expression.

### 3.2. GhZFP8 Positively Regulates the Elongation of Cotton Fibers

The expression of *GhZFP8* was low in the ovules of the fuzzless-lintless mutant at −1, 0, 1 DPA and in the wild type at −1 DPA, and then remarkably increased in the wild type at 0, 1 DPA [14]. In this study, *GhZFP8* had the highest level of fibers at 3 DPA, when cotton fibers begin to initiate and elongate. Meanwhile, the overexpression of *GhZFP8* enhanced the initiation of trichomes in *Arabidopsis*. Many of the key genes involved in trichome development have homologous genes in many plant species and similar biological functions. In cotton, *GhMYB1*, *GaMYB2*, *GhTTG1*, and *GhTTG3* regulate the development of the cotton fiber [31,32,33,34]. In this study, the interference of *GhZFP8* inhibited the fiber elongation. DAP-seq is an effective technology to identify the target genes of transcription factors in vitro, by which the GhZFP8 recognition sites in the putative target genes were identified in JM14. Previously studies have shown that photosynthesis is closely related to fiber development, and the overexpression or inhibition of some genes involved in photosynthesis could also change the quality or yield of cotton fiber [35]. Meanwhile, many regulators could play an important role in both photosynthesis and the development of cotton fiber at the same time [36,37]. GhZFP8 could bind to many photosynthesis-related genes associated with the biosynthesis of carbohydrates, including glucose and sucrose, which promote the initiation and elongation of fibers.

The development of cotton fiber is regulated by the synthesis or transport of biomass, such as fatty acids, sucrose, cellulose, etc. [38,39,40]. In this study, some GhZFP8-binding genes functioning on biosynthetic processes were identified, such as cellulose synthase, acyl-CoA oxidase, very-long-chain enoyl-CoA reductase, trehalose-phosphatase, etc. Meanwhile, calcium is a secondary signaling molecule, which plays an important role in regulating cotton fiber development [41,42]. Many genes encoding the key components of Ca^2+^ signal transduction, including calmodulin-binding protein, calcium-binding protein, Ca^2+^-dependent kinase, etc., were identified as GhZFP8′s putative target genes. Our result has illustrated that GhZFP8 has multiple functions in the development of cotton.

## 4. Materials and Methods

### 4.1. Plant Materials and Growth Conditions

Four cotton cultivars (*Gossypium hirsutum*) were used in this study, including Xinluzao33, Jinmian11 (J), 14m7-4 (H), and Jiaman14 (JM14). Cotton seeds of Xinluzao33 were sown in soil and grown under normal conditions for about twenty days. After the third true leaf emerged, uniform seedlings were transferred to the nutrient solution for two days. All of the seedlings were harvested for RNA extraction. The cotton seeds were then planted in the field. Their flowers were tagged on the day of anthesis. The fibers were collected on 3, 6, 9, 12, 15, 18, 21, 24, 27, and 30 days post-anthesis (DPA). The flowers were carefully excised from the developing bolls when the plants began to blossom. The leaves, stems, and roots were harvested from 25-day-old seedlings growing in pots filled with soil. The samples were frozen with liquid nitrogen and stored at −80 °C for total RNA extraction.

*Arabidopsis* (Col-0) was used for most experiments in this study. For phenotypic analysis, the plants were grown in cycles of 12 h of light and 12 h of darkness (22 °C). The number of trichomes on the siliques was monitored by counting all trichomes on the siliques’ surfaces. The total trichome number on the fourth rosette leaf was measured after four weeks of culturing. Trichome production on the sepals was evaluated by counting the trichomes on ten flowers per plant. The leaves were harvested from four-week-old plants for RNA extraction and phytohormone measurement to detect the gene expression and hormone content. SigmaPlot10 (v10.0) was used to draw the bar chart and GraphPad Prism 8 (v8.0.2) was used to analyze the data’s significance.

### 4.2. Construction of RNAi Vector and Generation of RNAi Transgenic Cotton

To construct the RNAi vector, the sequence of GhZFP8 (Gene accession XM_041111365 and Protein accession XP_040967299) was isolated from the JM14 cDNA using specific primers and then induced into a pBWA(V)HS vector by homologous recombination and seamless cloning of the golden gate. The pBWA(V)HS-GhZFP8 RNAi vector was transferred into *Agrobacterium tumefaciens* LBA4404 after sequencing. To generate RNAi transgenic plants, hypocotyls of JM14 were infected with LBA4404 harboring the *GhZFP8* RNAi vector. The transgenic cotton plants were characterized by PCR using genomic DNA and specific primers, and then the transcription of *GhZFP8* in the transgenic plants was detected by qPCR using cDNA as a template.

### 4.3. RNA Extraction and Quantitative PCR

To check the expression pattern of *GhZFP8*, total RNA was extracted from the roots, stems, leaves, flowers, and cotton fibers and stored at –80 °C. The cDNA synthesis was performed with M-MLV reverse transcriptase, according to the manufacturer’s instructions. Real-time PCR was performed to determine the gene expression with the gene-specific primers, and the cotton *UBI* gene was used as the internal control (Table S1). The qPCR conditions were as follows: pre-incubation at 94 °C for 1 min was followed by 45 cycles of 15 s of denaturing 95 °C, 20 s of annealing at 55 °C, and 30 s of extension at 72 °C. The final step was conducted at 72 °C for 10 min. Quantification was performed using the 2^−ΔΔCT^ method. The means of the three biological experiments were calculated as the expression level, and the expression assay was repeated three times, with each assay being performed with three independent technical repeats.

### 4.4. Analysis of the Correlation between GhZFP8 and the Development of Cotton Fiber

The cotton fiber of J02 and ZRI015, which generate long and short fibers, respectively [43], were harvested on 0, 3, 5, 10, 15, 20, and 25 DPA. The RNA-sequence was performed to obtain the transcriptomic data [44]. The cotton fiber of 251NIL was harvested on 15 DPA, and the average values of the fiber length (FL), fiber micronaire value (MV), fiber elongation (FE), fiber strength (FS), and fiber uniformity (FU) were measured. Then, an RNA sequence was performed to obtain the transcriptomic data [45]. The lme function in the R package lme4 was used to calculate the best linear unbiased predictive value (BLUP) of the upper FL, MV, FE, FS, and FU of 251 upland natural cotton populations, which were grown in four different places in two years, and the correlation analysis was conducted with the expression amount of the candidate genes in the 251 populations at 15 DPA.

### 4.5. Subcellular Localization of GhZFP8

The coding sequence of *GhZFP8* was cloned into a pBWA(V)HS-green fluorescent protein (GFP) vector with a homologous recombination and the gold gate method using specific primers (Supplementary Materials Table S1). To decrease the influence of GFP, a linker was inserted between the coding sequence of *GhZFP8* and GFP. Then, the pBWA(V)KS-*GhZFP8*-linker-GFP vector was transferred into the *Agrobacterium tumefaciens* strain EHA105. The cultures were diluted to OD600 = 1.5 and infiltrated into young tobacco (*Nicotiana benthamiana*) leaves. After infiltration, the tobacco plants were cultured for three days to induce the transient expression of the *GhZFP8*-GFP fusion gene. Then, the cells of the tobacco leaves were observed under a confocal microscope.

### 4.6. Measurement of Phytohormone in Arabidopsis

The sample preparation and extraction were performed as follows: Approximately 100 mg of frozen plant material samples were extracted in 1 mL of ice-cold, 50% aqueous ACN (VV). The samples were sonicated for 3 min at 4 °C and subsequently extracted using a benchtop laboratory rotator for 30 min at 15 rpm at 4 °C. After centrifugation (10 min, 12,000 rpm, 4 °C), the supernatant was transferred to clean plastic microtubes. All samples were purified using C18 reversed-phase, polymer-based, solid-phase extraction (RP-SPE) cartridges, washed with 1 mL of MeOH and 1 mL of deionized water, and then equilibrated with 50% aqueous ACN (V/Vl). After loading a sample, the cartridge was then rinsed with 1 mL of 30% ACN (V/Vl), and this fraction was collected. After this single step (SPE), the samples were evaporated to dryness under a gentle stream of nitrogen and stored at −20 °C until the analysis. For UHPLC–ESI–MS/MS analysis, the samples were dissolved in 200 μL of 30% ACN (V/V) and transferred to insert-equipped vials. The samples were analyzed using an UPLC–Orbitrap-MS system (UPLC, Vanquish; MS, QE). The analytical conditions were as follows: UPLC: column, Waters HSS T3 (50 × 2.1 mm, 1.7 μm); column temperature, 40 °C; flow rate, 0.3 mL/min; injection volume, 2 μL; solvent system, water (0.1% Acetic acid) and acetonitrile (0.1% Acetic acid); gradient program, 90:10 V/V at 0 min, 90:10 V/V at 1.0 min, 90:10 V/V at 7.0 min, 90:10 V/V at 7.1 min, and 90:10 V/V at 9.0 min.

The HRMS data were recorded with a Q Exactive hybrid Q–Orbitrap mass spectrometer equipped with a heated ESI source (Thermo Fisher Scientific, Waltham, MA, USA) using SIM MS acquisition methods. The ESI source parameters were set as follows: spray voltage, −2.8 kV/3.0 kV; sheath gas pressure, 40 arb; aux gas pressure, 10 arb; sweep gas pressure, 0 arb; capillary temperature, 320 °C; and aux gas heater temperature, 350 °C. The data were acquired with the Q-Exactive using Xcalibur 4.1 (Thermo Fisher Scientific) and were processed using TraceFinder™ 4.1 Clinical (Thermo Fisher Scientific). The quantified data were output into excel format.

### 4.7. DNA Afffnity Puriffcation Sequencing Analysis

The raw data were saved in Fastq format, and the quality control of the target protein (TF) sample and negative control (Halo) sample was performed using Fastp (version 0.20.1) software, including the removal of connectors, duplicate sequences, and low-quality sequences, to obtain clean data. Afterwards, bowtie2 (version 2.4.2) was used to sequence align clean reads with the specified reference genome and obtain a bam format alignment file. After deduplicating the results from the unique comparison, MACS2 (version 2.2.7.1) was used for peak calling. The obtained bigwig format file was visualized and displayed using IGV software (v1.5). The peak was annotated using R package ChIP seeker. Motif analysis was performed using HOMER (http://homer.ucsd.edu/homer/download.html, v4.11.1, accessed on 23 September 2023). For experiments involving two or more experimental groups, the differences between the experimental groups could be analyzed. We used MAnorm software (v1.3.0) for differential analysis on samples without biological duplication. For samples with biological replicates, we used Diffbind software (v3.2.7) for differential analysis.

## 5. Conclusions

GhZFP8 is a nuclear- and cytoplasm-localized protein that regulates the growth and development of *Arabidopsis*. Furthermore, the content of phytohormones and the expression of many genes are changed in transgenic *Arabidopsis*. Interestingly, the interference of *GhZFP8* inhibited the expansion of bolls and the elongation of fibers. GhZFP8 could bind to the promoter, exon, intron, and intergenic region of the candidate genes involved in photosynthesis, signal transduction, and synthesis of biomass. All of these findings will aid in understanding the biological function of *GhZFP8* on the development of cotton.

## Data Availability

The data that support the findings of this study are available on request from the corresponding author.

## Supplementary Materials

The following supporting information can be downloaded at: https://www.mdpi.com/article/10.3390/plants13040492/s1, Figure S1: Expression pattern of trichome-related genes in GhZFP8 overexpressor Arabidopsis; Figure S2: Transcription level of GhZFP8 in RNAi plants; Table S1: Primers used in this study; Table S2: Fiber parameters of the four cultivars used in this study; Table S3: Analysis of GhZFP8-binding region in the target genes; Table S4: Annotation of binding peak in GhZFP8 target genes; Table S5: GO enrichment analysis of GhZFP8 target genes; Table S6: KEGG enrichment analysis of GhZFP8 target genes.
