# Supplementary material for: Zinc Finger Protein8 (GhZFP8) Regulates the Initiation of Trichomes in Arabidopsis and the Development of Fiber in Cotton
Source: Plants (Basel). 2024 Feb 8;13(4):492. doi: 10.3390/plants13040492 (PMC10892670; doi:10.3390/plants13040492)
Supplement: Supplementary file 1 [file plants-13-00492-s001.zip › Supplementary Figure legends.pdf]

Figure S1: Expression of genes related to trichome development in *GhZFP8* overexpressor *Arabidopsis*

(A-I) Expression of *GL1* (A), *TTG1* (B), *GL3* (C), EGL3(D), TTG1(E), WER1(F), GA1(G), GIS(H), GIS3(I) in *GhZFP8* overexpressor *Arabidopsis*. RNA was isolated from leaves of 21-day-old plants, qRT-PCR was performed to check the expression of genes involved in trichome development. The expression of *ACTIN2* was used as a reference gene. Data represent the mean± SD of three replicates.

Figure S2: Transcription level of GhZFP8 in RNAi plants.
