# Supplementary material for: Zinc Finger Protein8 (GhZFP8) Regulates the Initiation of Trichomes in Arabidopsis and the Development of Fiber in Cotton
Source: Plants (Basel). 2024 Feb 8;13(4):492. doi: 10.3390/plants13040492 (PMC10892670; doi:10.3390/plants13040492)
Supplement: Supplementary file 1 [file plants-13-00492-s001.zip › Table S3 Analysis of GhZFP8 binding region in target genes.html]

homer\_motif/GhZFPH4\_DAP-vs-GhZFPH4\_neg - Homer Known Motif Enrichment Results


# Homer Known Motif Enrichment Results (homer\_motif/GhZFPH4\_DAP-vs-GhZFPH4\_neg)

Homer *de novo* Motif Results  
Gene Ontology Enrichment Results  
Known Motif Enrichment Results (txt file)  
Total Target Sequences = 8762, Total Background Sequences = 40806

|  |  |  |  |  |  |  |  |  |  |  |  |
| --- | --- | --- | --- | --- | --- | --- | --- | --- | --- | --- | --- |
| Rank | Motif | Name | P-value | log P-pvalue | q-value (Benjamini) | # Target Sequences with Motif | % of Targets Sequences with Motif | # Background Sequences with Motif | % of Background Sequences with Motif | Motif File | SVG |
| 1 | T G A C C T A G A C T G T A G C C G A T A C T G A T G C C A T G A T C G A T C G A T C G T A G C C T G A T A G C G C T A A C T G C G T A A G C T C G T A C T G A | GATA:SCL(Zf,bHLH)/Ter119-SCL-ChIP-Seq(GSE18720)/Homer | 1e-18 | -4.198e+01 | 0.0000 | 92.0 | 1.05% | 146.9 | 0.36% | motif file (matrix) | svg |
| 2 | C T G A C T A G C T A G A C T G C A G T A C G T C G A T C G T A C A T G C A T G A C T G C G A T C A G T C G A T C G T A | AT4G12670(MYBrelated)/col-AT4G12670-DAP-Seq(GSE60143)/Homer | 1e-16 | -3.800e+01 | 0.0000 | 27.0 | 0.31% | 14.1 | 0.03% | motif file (matrix) | svg |
| 3 | C A T G G A C T T A C G G T C A G T A C G A T C G A C T A G C T A T C G T C G A T A C G T A G C | ERRg(NR)/Kidney-ESRRG-ChIP-Seq(GSE104905)/Homer | 1e-13 | -3.118e+01 | 0.0000 | 570.0 | 6.51% | 1940.3 | 4.71% | motif file (matrix) | svg |
| 4 | T A G C G C A T A G T C G A T C A T G C G A C T C T A G A C T G A C T G C T G A A C T G C T A G A G T C T G A C C G A T | GLIS3(Zf)/Thyroid-Glis3.GFP-ChIP-Seq(GSE103297)/Homer | 1e-10 | -2.512e+01 | 0.0000 | 319.0 | 3.64% | 1012.7 | 2.46% | motif file (matrix) | svg |
| 5 | T A G C G C T A A C T G C G T A A C G T C G T A C G T A T A C G T C A G T C G A | Gata1(Zf)/K562-GATA1-ChIP-Seq(GSE18829)/Homer | 1e-9 | -2.266e+01 | 0.0000 | 768.0 | 8.77% | 2877.4 | 6.98% | motif file (matrix) | svg |
| 6 | T A C G A C T G A G C T G T A C C G T A T C G A C T G A A C T G C A T G A C G T A G T C C G T A | COUP-TFII(NR)/K562-NR2F1-ChIP-Seq(Encode)/Homer | 1e-9 | -2.208e+01 | 0.0000 | 875.0 | 9.99% | 3342.4 | 8.11% | motif file (matrix) | svg |
| 7 | A G C T A T G C G A C T G C A T C G T A A G C T G T A C C G A T A T C G A G T C | Gata6(Zf)/HUG1N-GATA6-ChIP-Seq(GSE51936)/Homer | 1e-9 | -2.199e+01 | 0.0000 | 1265.0 | 14.44% | 5033.0 | 12.21% | motif file (matrix) | svg |
| 8 | T C A G T C A G A C G T G T A C G C T A T C A G C T G A A C T G A C T G A G C T A G T C C G T A | EAR2(NR)/K562-NR2F6-ChIP-Seq(Encode)/Homer | 1e-9 | -2.146e+01 | 0.0000 | 728.0 | 8.31% | 2728.9 | 6.62% | motif file (matrix) | svg |
| 9 | A G C T A G T C A T G C A G C T A C G T C G T A A C G T A G T C C G A T A T G C | Gata2(Zf)/K562-GATA2-ChIP-Seq(GSE18829)/Homer | 1e-9 | -2.077e+01 | 0.0000 | 852.0 | 9.72% | 3268.4 | 7.93% | motif file (matrix) | svg |
| 10 | G C A T C T A G G T A C A G T C C G A T A C T G C T A G C T A G G T A C G C T A | ZNF416(Zf)/HEK293-ZNF416.GFP-ChIP-Seq(GSE58341)/Homer | 1e-8 | -2.028e+01 | 0.0000 | 334.0 | 3.81% | 1120.5 | 2.72% | motif file (matrix) | svg |
| 11 | G T C A G C T A G C T A T C G A A T C G A C G T A G T C T C G A T C G A T G A C | WRKY40(WRKY)/colamp-WRKY40-DAP-Seq(GSE60143)/Homer | 1e-8 | -1.852e+01 | 0.0000 | 706.0 | 8.06% | 2686.8 | 6.52% | motif file (matrix) | svg |
| 12 | G C A T A C G T A G T C A G C T C T G A A C T G C G T A C G T A T A C G T G A C G C A T G C A T G A T C G C A T C G T A | HSFB3(HSF)/colamp-HSFB3-DAP-Seq(GSE60143)/Homer | 1e-7 | -1.789e+01 | 0.0000 | 155.0 | 1.77% | 452.3 | 1.10% | motif file (matrix) | svg |
| 13 | G C T A G C A T G A C T G C A T T C A G G T A C G C T A G C A T C T G A G C T A T A G C G C T A C T G A C G A T C T A G | OCT4-SOX2-TCF-NANOG(POU,Homeobox,HMG)/mES-Oct4-ChIP-Seq(GSE11431)/Homer | 1e-7 | -1.718e+01 | 0.0000 | 363.0 | 4.14% | 1274.2 | 3.09% | motif file (matrix) | svg |
| 14 | T C A G C T A G C T A G C T A G T C A G T C G A C T G A C G A T A G T C G A T C A G T C T G A C | NFkB-p50,p52(RHD)/Monocyte-p50-ChIP-Chip(Schreiber\_et\_al.)/Homer | 1e-7 | -1.710e+01 | 0.0000 | 37.0 | 0.42% | 61.3 | 0.15% | motif file (matrix) | svg |
| 15 | C G T A C G T A C G T A C G T A C G T A A C T G A C T G A G T C | dof42(C2C2dof)/col-dof42-DAP-Seq(GSE60143)/Homer | 1e-7 | -1.666e+01 | 0.0000 | 803.0 | 9.17% | 3138.0 | 7.62% | motif file (matrix) | svg |
| 16 | A T G C G C A T C G A T G A T C A G C T C T G A A C T G C G T A C G T A T C A G T G A C C G A T G C A T G A T C C G A T | HSF21(HSF)/col-HSF21-DAP-Seq(GSE60143)/Homer | 1e-7 | -1.648e+01 | 0.0000 | 185.0 | 2.11% | 576.2 | 1.40% | motif file (matrix) | svg |
| 17 | C G T A C G T A G C A T A C T G C G T A A G C T C T G A C G T A T A C G C T G A | ELT-3(Gata)/cElegans-L1-ELT3-ChIP-Seq(modEncode)/Homer | 1e-7 | -1.644e+01 | 0.0000 | 900.0 | 10.27% | 3563.7 | 8.65% | motif file (matrix) | svg |
| 18 | G C A T C G A T A T G C A G C T T C G A T A C G G C T A C G T A C A T G T G A C G C A T C G A T A G T C A G C T C G T A | AT3G09735(S1Falike)/col-AT3G09735-DAP-Seq(GSE60143)/Homer | 1e-7 | -1.614e+01 | 0.0000 | 505.0 | 5.76% | 1877.8 | 4.56% | motif file (matrix) | svg |
| 19 | T C G A T G C A C A G T T C G A G A T C A G T C C G T A C G T A A C T G A G T C C G T A C G T A T C A G C G A T A G T C | AT5G25475(ABI3VP1)/col-AT5G25475-DAP-Seq(GSE60143)/Homer | 1e-6 | -1.519e+01 | 0.0000 | 950.0 | 10.84% | 3810.5 | 9.25% | motif file (matrix) | svg |
| 20 | C T G A C A G T C T G A A G T C C T A G G A C T A T C G G T A C | HIF-1b(HLH)/T47D-HIF1b-ChIP-Seq(GSE59937)/Homer | 1e-6 | -1.436e+01 | 0.0000 | 851.0 | 9.71% | 3397.6 | 8.25% | motif file (matrix) | svg |
| 21 | A G C T G A C T A C G T A C T G A C G T A G T C A C T G A C G T G C A T C G A T | AtIDD11(C2H2)/colamp-AtIDD11-DAP-Seq(GSE60143)/Homer | 1e-6 | -1.413e+01 | 0.0000 | 400.0 | 4.57% | 1470.9 | 3.57% | motif file (matrix) | svg |
| 22 | A C G T G A C T T A G C C G T A C T G A C A T G C T A G G A C T G A T C C G T A | Nr5a2(NR)/Pancreas-LRH1-ChIP-Seq(GSE34295)/Homer | 1e-6 | -1.392e+01 | 0.0000 | 393.0 | 4.49% | 1444.8 | 3.51% | motif file (matrix) | svg |
| 23 | A G T C C G A T C T G A C G T A A C G T C A G T T C A G T G A C | Isl1(Homeobox)/Neuron-Isl1-ChIP-Seq(GSE31456)/Homer | 1e-5 | -1.285e+01 | 0.0001 | 2758.0 | 31.48% | 12051.9 | 29.25% | motif file (matrix) | svg |
| 24 | C A T G G T A C G A T C A C T G A C G T A G C T C G T A C G T A | AT3G10030(Trihelix)/colamp-AT3G10030-DAP-Seq(GSE60143)/Homer | 1e-5 | -1.242e+01 | 0.0002 | 817.0 | 9.33% | 3295.1 | 8.00% | motif file (matrix) | svg |
| 25 | G T C A T G C A T G C A G C T A C G T A G C T A G C T A G C T A | REM19(REM)/colamp-REM19-DAP-Seq(GSE60143)/Homer | 1e-5 | -1.204e+01 | 0.0002 | 1017.0 | 11.61% | 4187.5 | 10.16% | motif file (matrix) | svg |
| 26 | A T G C A C T G C G A T T C A G A T G C C G T A C T G A T G C A C T G A G A C T A C T G G T C A | ABF1/SacCer-Promoters/Homer | 1e-5 | -1.193e+01 | 0.0003 | 299.0 | 3.41% | 1084.4 | 2.63% | motif file (matrix) | svg |
| 27 | G A C T A C G T A C G T A C T G A C G T A G T C G C A T A G C T G C A T G C A T G A C T A G C T | SGR5(C2H2)/colamp-SGR5-DAP-Seq(GSE60143)/Homer | 1e-5 | -1.159e+01 | 0.0003 | 673.0 | 7.68% | 2686.5 | 6.52% | motif file (matrix) | svg |
| 28 | C G T A C T G A C T A G C T G A A G T C G C T A C G A T A T C G G A C T G A T C A G T C C T G A C T A G C T A G A G T C G C T A C G A T C T A G G A T C G A T C | p73(p53)/Trachea-p73-ChIP-Seq(PRJNA310161)/Homer | 1e-4 | -1.149e+01 | 0.0004 | 38.0 | 0.43% | 81.9 | 0.20% | motif file (matrix) | svg |
| 29 | C G T A G A T C A G C T A C G T A C G T A C T G C G T A G T A C A G C T G C T A C G A T C G A T C G A T G C A T G C T A | WRKY18(WRKY)/col-WRKY18-DAP-Seq(GSE60143)/Homer | 1e-4 | -1.103e+01 | 0.0006 | 1748.0 | 19.95% | 7505.1 | 18.21% | motif file (matrix) | svg |
| 30 | C G T A G A T C C A T G G C A T G A C T C T A G T C G A T A G C A G C T G C A T | WRKY55(WRKY)/col-WRKY55-DAP-Seq(GSE60143)/Homer | 1e-4 | -1.090e+01 | 0.0006 | 1241.0 | 14.17% | 5221.6 | 12.67% | motif file (matrix) | svg |
| 31 | C T G A C G A T C A T G A T C G G C A T C A T G G C T A A G T C | ASHR1(ND)/col-ASHR1-DAP-Seq(GSE60143)/Homer | 1e-4 | -1.075e+01 | 0.0007 | 984.0 | 11.23% | 4078.2 | 9.90% | motif file (matrix) | svg |
| 32 | A C G T G A C T A T G C G C T A C T G A C T A G A C T G G A C T A G T C C G T A | Nr5a2(NR)/mES-Nr5a2-ChIP-Seq(GSE19019)/Homer | 1e-4 | -1.058e+01 | 0.0008 | 263.0 | 3.00% | 955.6 | 2.32% | motif file (matrix) | svg |
| 33 | T A C G C T A G A T G C G A T C G T A C A G T C C T A G A G T C A G T C A G T C G T A C A G T C | Sp1(Zf)/Promoter/Homer | 1e-4 | -1.056e+01 | 0.0008 | 22.0 | 0.25% | 37.2 | 0.09% | motif file (matrix) | svg |
| 34 | T C A G C G T A C G A T C G A T A G T C T A G C T A C G C A G T G A C T C T G A | MYB105(MYB)/colamp-MYB105-DAP-Seq(GSE60143)/Homer | 1e-4 | -1.054e+01 | 0.0008 | 1090.0 | 12.44% | 4557.8 | 11.06% | motif file (matrix) | svg |
| 35 | G C A T G C A T G A T C G A C T T C G A T C A G G C T A C G T A A C T G G T A C G C A T G C A T A G T C A G C T C G T A | HSF7(HSF)/colamp-HSF7-DAP-Seq(GSE60143)/Homer | 1e-4 | -1.027e+01 | 0.0010 | 244.0 | 2.79% | 881.7 | 2.14% | motif file (matrix) | svg |
| 36 | A C T G T G A C G T A C C G T A A G T C T A C G A C G T A C T G G T C A A G T C | NPAS2(bHLH)/Liver-NPAS2-ChIP-Seq(GSE39860)/Homer | 1e-4 | -1.013e+01 | 0.0011 | 623.0 | 7.11% | 2503.5 | 6.08% | motif file (matrix) | svg |
| 37 | G T A C A G C T C T G A G T C A T G A C A C T G C T A G G T A C G A T C G A C T | MYB56(MYB)/colamp-MYB56-DAP-Seq(GSE60143)/Homer | 1e-4 | -1.011e+01 | 0.0011 | 1180.0 | 13.47% | 4974.5 | 12.07% | motif file (matrix) | svg |
| 38 | T A C G A T G C G C T A A C T G C G T A A C G T C G T A C T G A T A C G T C G A | Gata4(Zf)/Heart-Gata4-ChIP-Seq(GSE35151)/Homer | 1e-4 | -9.848e+00 | 0.0014 | 1360.0 | 15.52% | 5793.6 | 14.06% | motif file (matrix) | svg |
| 39 | C T G A A T C G G T A C C T G A A G T C A G T C A C T G C G T A A G T C C T G A | TINY(AP2EREBP)/col-TINY-DAP-Seq(GSE60143)/Homer | 1e-4 | -9.419e+00 | 0.0021 | 185.0 | 2.11% | 651.9 | 1.58% | motif file (matrix) | svg |
| 40 | C T G A T G C A A G T C A C T G A C G T T C A G C G A T G C A T G C A T G A T C G C A T G A T C G T C A A G T C A C T G | ANAC094(NAC)/col-ANAC094-DAP-Seq(GSE60143)/Homer | 1e-4 | -9.405e+00 | 0.0021 | 206.0 | 2.35% | 737.9 | 1.79% | motif file (matrix) | svg |
| 41 | C T G A C G A T C A G T C A G T C G T A C G T A A C G T C A T G C T A G A T C G | EGL-5(Homeobox)/cElegans-L3-EGL5-ChIP-Seq(modEncode)/Homer | 1e-4 | -9.268e+00 | 0.0023 | 2415.0 | 27.57% | 10632.4 | 25.80% | motif file (matrix) | svg |
| 42 | C G T A C G T A C T G A C A G T T A G C C G T A G A T C C T A G G C A T C A T G G T A C G A C T | BIM2(bHLH)/col-BIM2-DAP-Seq(GSE60143)/Homer | 1e-4 | -9.242e+00 | 0.0023 | 660.0 | 7.53% | 2688.9 | 6.53% | motif file (matrix) | svg |
| 43 | A C T G C G T A C G T A A C G T G T A C G A C T C G T A C G A T C T G A C T G A | AT1G49560(G2like)/colamp-AT1G49560-DAP-Seq(GSE60143)/Homer | 1e-3 | -8.994e+00 | 0.0029 | 1965.0 | 22.43% | 8579.5 | 20.82% | motif file (matrix) | svg |
| 44 | G C T A C G T A C G T A C G T A C G A T C G A T A G T C G T A C A C T G A C G T A C G T C G T A A G C T G C T A G C A T | MYB113(MYB)/col-MYB113-DAP-Seq(GSE60143)/Homer | 1e-3 | -8.952e+00 | 0.0030 | 1018.0 | 11.62% | 4287.7 | 10.41% | motif file (matrix) | svg |
| 45 | C T A G C A T G T G C A A C G T A G T C C G T A C A T G T C A G A C G T A C G T G C T A A G T C | Six1(Homeobox)/Myoblast-Six1-ChIP-Chip(GSE20150)/Homer | 1e-3 | -8.835e+00 | 0.0033 | 319.0 | 3.64% | 1219.6 | 2.96% | motif file (matrix) | svg |
| 46 | T A C G T A G C C A T G C A G T A C G T C T A G C G T A A G T C G A C T G C A T G C A T C A G T | WRKY11(WRKY)/col-WRKY11-DAP-Seq(GSE60143)/Homer | 1e-3 | -8.741e+00 | 0.0035 | 155.0 | 1.77% | 538.9 | 1.31% | motif file (matrix) | svg |
| 47 | G A C T C T G A A G T C A G T C A C T G C G T A A G T C C T G A | bHLH10(bHLH)/colamp-bHLH10-DAP-Seq(GSE60143)/Homer | 1e-3 | -8.702e+00 | 0.0036 | 213.0 | 2.43% | 776.3 | 1.88% | motif file (matrix) | svg |
| 48 | C G T A T A G C A G T C C T A G C A G T C T A G C T G A G T A C G C A T T C G A C G T A G C A T A G C T C T A G T C G A | PAX3:FKHR-fusion(Paired,Homeobox)/Rh4-PAX3:FKHR-ChIP-Seq(GSE19063)/Homer | 1e-3 | -8.609e+00 | 0.0038 | 318.0 | 3.63% | 1219.9 | 2.96% | motif file (matrix) | svg |
| 49 | T C G A T G C A A C T G G T C A A G T C A G T C A G T C A G T C A G C T T G A C | LRF(Zf)/Erythroblasts-ZBTB7A-ChIP-Seq(GSE74977)/Homer | 1e-3 | -8.524e+00 | 0.0041 | 431.0 | 4.92% | 1706.2 | 4.14% | motif file (matrix) | svg |
| 50 | G C A T C G T A G C A T C G T A T C G A C G T A C T G A A C T G C G T A C G T A C G T A A C G T A C T G G T C A G C A T | AT2G31460(REMB3)/col-AT2G31460-DAP-Seq(GSE60143)/Homer | 1e-3 | -8.510e+00 | 0.0041 | 497.0 | 5.67% | 1993.0 | 4.84% | motif file (matrix) | svg |
| 51 | A T C G T G C A G A T C C T A G A C G T A T C G C G T A A G T C T C A G A C T G T C A G G C T A | Knotted(Homeobox)/Corn-KN1-ChIP-Seq(GSE39161)/Homer | 1e-3 | -8.346e+00 | 0.0047 | 1016.0 | 11.60% | 4299.0 | 10.43% | motif file (matrix) | svg |
| 52 | G A C T G C A T A C G T A C G T A C T G C G T A A G T C A G C T C G A T A T C G G C A T A C T G C G A T C T A G C G T A | WRKY50(WRKY)/col-WRKY50-DAP-Seq(GSE60143)/Homer | 1e-3 | -8.138e+00 | 0.0057 | 1143.0 | 13.05% | 4878.7 | 11.84% | motif file (matrix) | svg |
| 53 | G C T A T A G C A G C T A T C G G T C A C G T A G C T A A T G C G A T C C T G A | IRF4(IRF)/GM12878-IRF4-ChIP-Seq(GSE32465)/Homer | 1e-3 | -8.122e+00 | 0.0057 | 579.0 | 6.61% | 2362.6 | 5.73% | motif file (matrix) | svg |
| 54 | C A T G G T A C A C T G G T C A A G C T T A C G T G C A A T C G T G A C C A G T | TOD6?/SacCer-Promoters/Homer | 1e-3 | -8.066e+00 | 0.0058 | 146.0 | 1.67% | 510.5 | 1.24% | motif file (matrix) | svg |
| 55 | A T G C A T C G A T G C A T C G A T G C A T C G A T G C A T C G A T G C A T C G | SeqBias: CG-repeat | 1e-3 | -7.928e+00 | 0.0066 | 249.0 | 2.84% | 939.9 | 2.28% | motif file (matrix) | svg |
| 56 | C G A T T G C A G T A C C G T A A G T C C T A G G A C T C A T G | NPAS(bHLH)/Liver-NPAS-ChIP-Seq(GSE39860)/Homer | 1e-3 | -7.927e+00 | 0.0066 | 1137.0 | 12.98% | 4859.0 | 11.79% | motif file (matrix) | svg |
| 57 | C G T A T C G A T A C G A G T C C G T A A G T C A C T G A C G T A C T G A G C T T C A G C G A T | Pho4(bHLH)/Yeast-Pho4-ChIP-Seq(GSE29506)/Homer | 1e-3 | -7.816e+00 | 0.0071 | 77.0 | 0.88% | 240.2 | 0.58% | motif file (matrix) | svg |
| 58 | C G T A C G T A A G C T G A C T T G C A G T C A A C G T A G C T C T G A T C A G | Lhx3(Homeobox)/Neuron-Lhx3-ChIP-Seq(GSE31456)/Homer | 1e-3 | -7.772e+00 | 0.0073 | 3076.0 | 35.11% | 13769.8 | 33.42% | motif file (matrix) | svg |
| 59 | C G T A A G T C T G A C A G C T A C G T C G T A A C G T A G T C | At5g05790(MYBrelated)/col-At5g05790-DAP-Seq(GSE60143)/Homer | 1e-3 | -7.721e+00 | 0.0076 | 1474.0 | 16.82% | 6395.3 | 15.52% | motif file (matrix) | svg |
| 60 | G A T C C G T A G A C T C T A G G A T C C T G A G A C T C T G A G A C T C T A G G A T C C T G A G A C T C T G A G A C T | OCT:OCT(POU,Homeobox)/NPC-OCT6-ChIP-Seq(GSE43916)/Homer | 1e-3 | -7.694e+00 | 0.0076 | 244.0 | 2.79% | 922.7 | 2.24% | motif file (matrix) | svg |
| 61 | C G A T G C T A G C T A G C A T G C T A C G T A A G T C A C G T A C G T A C G T C G A T A G C T | At5g62940(C2C2dof)/col-At5g62940-DAP-Seq(GSE60143)/Homer | 1e-3 | -7.685e+00 | 0.0076 | 4362.0 | 49.79% | 19784.9 | 48.02% | motif file (matrix) | svg |
| 62 | T A C G T A C G G T A C A T C G A C T G T A C G T C G A C T G A T C G A A T C G | E2F6(E2F)/Hela-E2F6-ChIP-Seq(GSE31477)/Homer | 1e-3 | -7.652e+00 | 0.0077 | 97.0 | 1.11% | 319.1 | 0.77% | motif file (matrix) | svg |
| 63 | C T A G T G A C G A C T A T C G T C G A A G T C C T A G C A G T C T A G A T C G G T A C T C G A | O2(bZIP)/Corn-O2-ChIP-Seq(GSE63991)/Homer | 1e-3 | -7.616e+00 | 0.0079 | 103.0 | 1.18% | 343.1 | 0.83% | motif file (matrix) | svg |
| 64 | A C T G C G T A A C G T C G T A C T G A A C T G T C A G G C A T | At3g11280(MYBrelated)/col-At3g11280-DAP-Seq(GSE60143)/Homer | 1e-3 | -7.396e+00 | 0.0096 | 1408.0 | 16.07% | 6109.5 | 14.83% | motif file (matrix) | svg |
| 65 | G A T C C T G A A G T C C G A T C G A T G A T C A G T C A C T G A T C G A G C T | Elk4(ETS)/Hela-Elk4-ChIP-Seq(GSE31477)/Homer | 1e-3 | -7.374e+00 | 0.0097 | 450.0 | 5.14% | 1816.1 | 4.41% | motif file (matrix) | svg |
| 66 | C T G A T A G C G C T A C G A T A T C G A G T C G A T C G A T C C T A G T C A G T C A G G T A C G C T A C A G T | p53(p53)/mES-cMyc-ChIP-Seq(GSE11431)/Homer | 1e-3 | -7.237e+00 | 0.0110 | 9.0 | 0.10% | 11.5 | 0.03% | motif file (matrix) | svg |
| 67 | T A G C A T G C C G T A A G T C C T A G G A C T T A C G T C A G A G C T G C T A | PIF7(bHLH)/col-PIF7-DAP-Seq(GSE60143)/Homer | 1e-3 | -7.174e+00 | 0.0115 | 69.0 | 0.79% | 215.5 | 0.52% | motif file (matrix) | svg |
| 68 | A C T G C T A G G A T C C G T A C T A G A G T C T G C A G A C T C G T A A C G T T C A G A G T C A C G T C T G A A G T C A G T C G A T C C G T A T C A G T A C G | EBNA1(EBV-virus)/Raji-EBNA1-ChIP-Seq(GSE30709)/Homer | 1e-3 | -7.096e+00 | 0.0123 | 8.0 | 0.09% | 9.5 | 0.02% | motif file (matrix) | svg |
| 69 | C A T G A G C T T A C G G T C A G T A C T A G C A G C T G A C T A T C G T C G A | Esrrb(NR)/mES-Esrrb-ChIP-Seq(GSE11431)/Homer | 1e-3 | -7.033e+00 | 0.0129 | 424.0 | 4.84% | 1711.6 | 4.15% | motif file (matrix) | svg |
| 70 | G C A T G C A T G C A T A T G C A G C T T C G A T A C G G C T A C G T A C A T G G T A C G C A T G C A T A G T C A G C T | HSFA6B(HSF)/colamp-HSFA6B-DAP-Seq(GSE60143)/Homer | 1e-3 | -7.012e+00 | 0.0130 | 658.0 | 7.51% | 2744.4 | 6.66% | motif file (matrix) | svg |
| 71 | T A G C G T C A G A C T T A G C G T C A G A C T A G T C G C T A G A C T G A T C | ZML2(C2C2gata)/col-ZML2-DAP-Seq(GSE60143)/Homer | 1e-3 | -7.005e+00 | 0.0130 | 354.0 | 4.04% | 1407.1 | 3.41% | motif file (matrix) | svg |
| 72 | A C T G C A G T A C G T C G T A C G T A A C G T A C T G C T G A | Nkx6.1(Homeobox)/Islet-Nkx6.1-ChIP-Seq(GSE40975)/Homer | 1e-3 | -6.971e+00 | 0.0131 | 4429.0 | 50.55% | 20144.5 | 48.89% | motif file (matrix) | svg |
| 73 | C T G A G A T C G C A T A C T G C G T A A C G T C G T A C G T A T A C G T C G A | PQM-1(?)/cElegans-L3-ChIP-Seq(modEncode)/Homer | 1e-3 | -6.925e+00 | 0.0135 | 618.0 | 7.05% | 2569.6 | 6.24% | motif file (matrix) | svg |
| 74 | C T G A A C T G C G T A C G T A A C G T G T A C G A C T G C A T G C A T C G A T | AT4G37180(G2like)/col-AT4G37180-DAP-Seq(GSE60143)/Homer | 1e-2 | -6.893e+00 | 0.0138 | 1556.0 | 17.76% | 6807.5 | 16.52% | motif file (matrix) | svg |
| 75 | A G T C A G T C C T G A A G T C A G T C A C T G C G T A A G T C C T G A T C G A G C A T G A T C C G A T C G A T A C T G | AT3G60490(AP2EREBP)/colamp-AT3G60490-DAP-Seq(GSE60143)/Homer | 1e-2 | -6.744e+00 | 0.0158 | 205.0 | 2.34% | 774.1 | 1.88% | motif file (matrix) | svg |
| 76 | C G T A C T G A C G T A C T A G T C G A C T A G A C T G C G T A C G T A T A C G A G C T A T C G | SpiB(ETS)/OCILY3-SPIB-ChIP-Seq(GSE56857)/Homer | 1e-2 | -6.738e+00 | 0.0158 | 162.0 | 1.85% | 594.7 | 1.44% | motif file (matrix) | svg |
| 77 | C T A G T A C G A G T C G T A C T C G A A C G T G T C A G C T A G C T A C G A T A G T C G C T A | HOXA9(Homeobox)/HSC-Hoxa9-ChIP-Seq(GSE33509)/Homer | 1e-2 | -6.643e+00 | 0.0170 | 1103.0 | 12.59% | 4757.4 | 11.55% | motif file (matrix) | svg |
| 78 | T G A C C T G A A G T C C T A G G A C T A C T G C G A T C G A T G A C T G T A C C G T A A G T C C T A G A G C T A C T G | bHLH18(bHLH)/col-bHLH18-DAP-Seq(GSE60143)/Homer | 1e-2 | -6.413e+00 | 0.0211 | 20.0 | 0.23% | 44.6 | 0.11% | motif file (matrix) | svg |
| 79 | A C T G A C G T C A T G A T C G A T C G T G A C A C T G A T C G A T C G T G C A C T G A C G T A | E2F3(E2F)/MEF-E2F3-ChIP-Seq(GSE71376)/Homer | 1e-2 | -6.321e+00 | 0.0229 | 165.0 | 1.88% | 613.8 | 1.49% | motif file (matrix) | svg |
| 80 | T C G A T C A G T C G A A C T G C A T G A C G T A G T C C T G A | COUP-TFII(NR)/Artia-Nr2f2-ChIP-Seq(GSE46497)/Homer | 1e-2 | -6.282e+00 | 0.0235 | 921.0 | 10.51% | 3949.1 | 9.58% | motif file (matrix) | svg |
| 81 | G A T C T C G A A G T C C G A T C G A T A G T C A T G C A C T G A T C G G A C T | Elk1(ETS)/Hela-Elk1-ChIP-Seq(GSE31477)/Homer | 1e-2 | -6.058e+00 | 0.0291 | 304.0 | 3.47% | 1212.8 | 2.94% | motif file (matrix) | svg |
| 82 | C T A G G T A C C A T G G A C T C G A T C A T G G T C A G T A C G A C T C G A T C G A T C G A T | WRKY27(WRKY)/colamp-WRKY27-DAP-Seq(GSE60143)/Homer | 1e-2 | -6.033e+00 | 0.0294 | 1150.0 | 13.13% | 4997.0 | 12.13% | motif file (matrix) | svg |
| 83 | A G C T C G T A C G T A A G T C C T A G C T A G G T A C G A C T | MYB101(MYB)/colamp-MYB101-DAP-Seq(GSE60143)/Homer | 1e-2 | -6.030e+00 | 0.0294 | 2248.0 | 25.66% | 10035.3 | 24.35% | motif file (matrix) | svg |
| 84 | G A C T T C A G G C A T A G T C G C T A G A T C C T G A A C G T A G T C G T C A | Replumless(BLH)/Arabidopsis-RPL.GFP-ChIP-Seq(GSE78727)/Homer | 1e-2 | -6.022e+00 | 0.0294 | 1292.0 | 14.75% | 5644.0 | 13.70% | motif file (matrix) | svg |
| 85 | C T G A C T G A A G T C T A G C G A C T G C A T C T G A A G C T A G T C A G T C | At5g08520(MYBrelated)/colamp-At5g08520-DAP-Seq(GSE60143)/Homer | 1e-2 | -5.956e+00 | 0.0307 | 1459.0 | 16.65% | 6410.5 | 15.56% | motif file (matrix) | svg |
| 86 | C A G T A C T G T C A G T G C A G C T A A T G C T C G A A T C G G T C A T G C A | ZNF189(Zf)/HEK293-ZNF189.GFP-ChIP-Seq(GSE58341)/Homer | 1e-2 | -5.936e+00 | 0.0309 | 436.0 | 4.98% | 1793.7 | 4.35% | motif file (matrix) | svg |
| 87 | G A T C G C A T G C A T A G T C A G C T T C G A T A C G G C T A C G T A C T A G T G A C G C A T C G A T G A T C A G C T | HSFC1(HSF)/col-HSFC1-DAP-Seq(GSE60143)/Homer | 1e-2 | -5.931e+00 | 0.0309 | 242.0 | 2.76% | 947.1 | 2.30% | motif file (matrix) | svg |
| 88 | T A G C G T A C C T A G C A G T T C G A C G T A C G T A G C A T G A C T T G A C A G T C A C T G A T C G A G T C C T A G | AS2(LOBAS2)/col-AS2-DAP-Seq(GSE60143)/Homer | 1e-2 | -5.914e+00 | 0.0309 | 37.0 | 0.42% | 105.7 | 0.26% | motif file (matrix) | svg |
| 89 | C T G A C T G A C A T G A T C G A G C T A T C G G A C T C A T G C T G A G T C A | Tbr1(T-box)/Cortex-Tbr1-ChIP-Seq(GSE71384)/Homer | 1e-2 | -5.897e+00 | 0.0311 | 1523.0 | 17.38% | 6706.9 | 16.28% | motif file (matrix) | svg |
| 90 | G C T A G A C T G A C T T G C A C G T A A G T C C G T A T A G C G A T C G A C T | Eomes(T-box)/H9-Eomes-ChIP-Seq(GSE26097)/Homer | 1e-2 | -5.889e+00 | 0.0311 | 2308.0 | 26.34% | 10321.0 | 25.05% | motif file (matrix) | svg |
| 91 | C G T A C A T G C A T G A C T G C T A G T C G A G C A T C G A T A G C T A G T C G A T C G T A C | NFkB-p65(RHD)/GM12787-p65-ChIP-Seq(GSE19485)/Homer | 1e-2 | -5.888e+00 | 0.0311 | 265.0 | 3.02% | 1047.3 | 2.54% | motif file (matrix) | svg |
| 92 | G C T A T G A C T G C A A T G C A C T G A G T C A C G T A G T C A G T C G C A T | MYB88(MYB)/col-MYB88-DAP-Seq(GSE60143)/Homer | 1e-2 | -5.723e+00 | 0.0358 | 572.0 | 6.53% | 2403.0 | 5.83% | motif file (matrix) | svg |
| 93 | C T G A T C A G G T A C T G C A A G T C C G T A A G T C A C T G A C G T A C T G | MNT(bHLH)/HepG2-MNT-ChIP-Seq(Encode)/Homer | 1e-2 | -5.616e+00 | 0.0394 | 680.0 | 7.76% | 2890.0 | 7.01% | motif file (matrix) | svg |
| 94 | C T A G T A C G G A T C G T A C C T G A A G C T T G C A G C T A C G T A G C A T G A T C G C T A | Hoxc9(Homeobox)/Ainv15-Hoxc9-ChIP-Seq(GSE21812)/Homer | 1e-2 | -5.576e+00 | 0.0405 | 840.0 | 9.59% | 3612.9 | 8.77% | motif file (matrix) | svg |
| 95 | C G A T C T G A C T A G G T A C A C T G A G T C C T A G A G T C | DPL-1(E2F)/cElegans-Adult-ChIP-Seq(modEncode)/Homer | 1e-2 | -5.524e+00 | 0.0423 | 227.0 | 2.59% | 891.1 | 2.16% | motif file (matrix) | svg |
| 96 | C T A G G A C T C T A G A C T G C T A G G C T A G A T C G A T C G A T C T C G A A G T C C G A T C G A T G C A T C G A T | At2g45680(TCP)/colamp-At2g45680-DAP-Seq(GSE60143)/Homer | 1e-2 | -5.480e+00 | 0.0437 | 23.0 | 0.26% | 58.7 | 0.14% | motif file (matrix) | svg |
| 97 | C T G A C G A T A C G T A C G T A C G T C T G A A G T C A C T G C G T A A C G T | ARF16(ARF)/col-ARF16-DAP-Seq(GSE60143)/Homer | 1e-2 | -5.474e+00 | 0.0437 | 372.0 | 4.25% | 1524.6 | 3.70% | motif file (matrix) | svg |
| 98 | A G C T C T A G T G A C C G T A A C G T C G A T A G T C A G T C C T G A C A T G | TEAD3(TEA)/HepG2-TEAD3-ChIP-Seq(Encode)/Homer | 1e-2 | -5.346e+00 | 0.0490 | 1015.0 | 11.59% | 4415.6 | 10.72% | motif file (matrix) | svg |
| 99 | C G T A C A T G A G T C G A C T T G C A C G T A A C G T A C G T C T G A T C A G | Lhx1(Homeobox)/EmbryoCarcinoma-Lhx1-ChIP-Seq(GSE70957)/Homer | 1e-2 | -5.338e+00 | 0.0490 | 2163.0 | 24.69% | 9685.9 | 23.51% | motif file (matrix) | svg |
| 100 | G A T C C T G A A G T C A G T C A C T G C G T A A G T C C T G A | ERF38(AP2EREBP)/col-ERF38-DAP-Seq(GSE60143)/Homer | 1e-2 | -5.307e+00 | 0.0499 | 276.0 | 3.15% | 1108.3 | 2.69% | motif file (matrix) | svg |
| 101 | T C G A C T G A C G T A G C A T C G A T A G C T T C G A T G C A T G C A G C A T | TSO1(CPP)/col-TSO1-DAP-Seq(GSE60143)/Homer | 1e-2 | -5.260e+00 | 0.0518 | 389.0 | 4.44% | 1605.6 | 3.90% | motif file (matrix) | svg |
| 102 | A T G C G A T C C G A T C T A G A C T G G C T A C G T A A G C T A C T G A G C T | TEAD2(TEA)/Py2T-Tead2-ChIP-Seq(GSE55709)/Homer | 1e-2 | -5.259e+00 | 0.0518 | 311.0 | 3.55% | 1262.2 | 3.06% | motif file (matrix) | svg |
| 103 | A T G C T A C G A G T C G A C T T G C A C T G A G A C T A C G T C T G A T C A G | LHX9(Homeobox)/Hct116-LHX9.V5-ChIP-Seq(GSE116822)/Homer | 1e-2 | -5.257e+00 | 0.0518 | 2298.0 | 26.23% | 10315.3 | 25.03% | motif file (matrix) | svg |
| 104 | G C A T C T G A G T C A G T A C C G T A A G T C C T A G A C G T A C T G C A G T G A C T C G T A G C T A C G T A G C A T | GT3a(Trihelix)/col-GT3a-DAP-Seq(GSE60143)/Homer | 1e-2 | -5.237e+00 | 0.0518 | 141.0 | 1.61% | 530.8 | 1.29% | motif file (matrix) | svg |
| 105 | C T A G C T G A C T A G C T G A C T A G C T G A C T A G C T G A C T A G C T G A | SeqBias: GA-repeat | 1e-2 | -5.226e+00 | 0.0518 | 4584.0 | 52.32% | 20996.7 | 50.96% | motif file (matrix) | svg |
| 106 | C G T A C T G A G T C A A C G T A G T C C G T A A G T C A C T G A C G T A C T G T G C A A G C T | bHLH74(bHLH)/col-bHLH74-DAP-Seq(GSE60143)/Homer | 1e-2 | -5.120e+00 | 0.0567 | 75.0 | 0.86% | 259.9 | 0.63% | motif file (matrix) | svg |
| 107 | C T A G A C T G C T A G T C A G T C A G T A C G C T A G A C T G | Maz(Zf)/HepG2-Maz-ChIP-Seq(GSE31477)/Homer | 1e-2 | -5.112e+00 | 0.0567 | 181.0 | 2.07% | 702.3 | 1.70% | motif file (matrix) | svg |
| 108 | C G T A A C G T A C G T A C G T A C G T A G T C A G T C C T G A A G C T A G C T | NFAT(RHD)/Jurkat-NFATC1-ChIP-Seq(Jolma\_et\_al.)/Homer | 1e-2 | -5.109e+00 | 0.0567 | 947.0 | 10.81% | 4117.8 | 9.99% | motif file (matrix) | svg |
| 109 | C G A T A C T G A G C T T G A C C T G A A G T C C T A G G C A T A T C G C G T A | SPCH(bHLH)/Seedling-SPCH-ChIP-Seq(GSE57497)/Homer | 1e-2 | -5.003e+00 | 0.0620 | 807.0 | 9.21% | 3487.4 | 8.46% | motif file (matrix) | svg |
| 110 | T C A G T C A G G C T A C G T A T A C G G A C T T C A G T C G A C T G A C G T A T A C G G A C T | IRF8(IRF)/BMDM-IRF8-ChIP-Seq(GSE77884)/Homer | 1e-2 | -4.990e+00 | 0.0623 | 346.0 | 3.95% | 1424.0 | 3.46% | motif file (matrix) | svg |
| 111 | T A C G T A G C G C T A C G A T C T A G A C G T C A G T C A G T G C T A A G T C G T C A G C A T | FOXK2(Forkhead)/U2OS-FOXK2-ChIP-Seq(E-MTAB-2204)/Homer | 1e-2 | -4.900e+00 | 0.0675 | 663.0 | 7.57% | 2841.8 | 6.90% | motif file (matrix) | svg |
| 112 | G A T C G T A C C G A T A C T G A C T G C G T A C G T A A C G T A C T G G A T C | TEAD(TEA)/Fibroblast-PU.1-ChIP-Seq(Unpublished)/Homer | 1e-2 | -4.900e+00 | 0.0675 | 619.0 | 7.07% | 2643.1 | 6.41% | motif file (matrix) | svg |
| 113 | G A T C G C A T A G T C A G C T G A T C A G C T G A T C G A C T G A T C G A C T A G T C A C G T G A T C A G C T G A T C | GAGA-repeat/SacCer-Promoters/Homer | 1e-2 | -4.822e+00 | 0.0717 | 3335.0 | 38.07% | 15171.6 | 36.82% | motif file (matrix) | svg |
| 114 | A G C T A G C T T A G C A T C G A G T C A C T G A T G C A T C G T C G A C T G A T C G A C T G A | E2F(E2F)/Hela-CellCycle-Expression/Homer | 1e-2 | -4.654e+00 | 0.0840 | 24.0 | 0.27% | 66.7 | 0.16% | motif file (matrix) | svg |
| 115 | C G T A C T A G G A C T G T C A G T C A C G T A A G T C C G T A T C G A T C G A T C G A C G T A C T G A C T A G G C T A C G T A T A G C C G T A C G A T C G T A | FOXA1:AR(Forkhead,NR)/LNCAP-AR-ChIP-Seq(GSE27824)/Homer | 1e-2 | -4.642e+00 | 0.0843 | 78.0 | 0.89% | 277.2 | 0.67% | motif file (matrix) | svg |
| 116 | A T G C G A T C C G T A C G T A A C G T A T G C C G T A A C G T C G A T C T A G A C T G G T A C | HAT1(Homeobox)/col-HAT1-DAP-Seq(GSE60143)/Homer | 1e-2 | -4.639e+00 | 0.0843 | 312.0 | 3.56% | 1283.3 | 3.11% | motif file (matrix) | svg |
| 117 | T C G A A G T C A C G T A C G T T C A G C A G T C T G A C T A G T C G A C G T A A T C G C G T A C G T A A C T G A G C T | NTM1(NAC)/col-NTM1-DAP-Seq(GSE60143)/Homer | 1e-2 | -4.614e+00 | 0.0853 | 469.0 | 5.35% | 1981.2 | 4.81% | motif file (matrix) | svg |
