# Supplementary figures and images for: Zinc Finger Protein8 (GhZFP8) Regulates the Initiation of Trichomes in Arabidopsis and the Development of Fiber in Cotton
Source: Plants (Basel). 2024 Feb 8;13(4):492. doi: 10.3390/plants13040492 (PMC10892670; doi:10.3390/plants13040492)

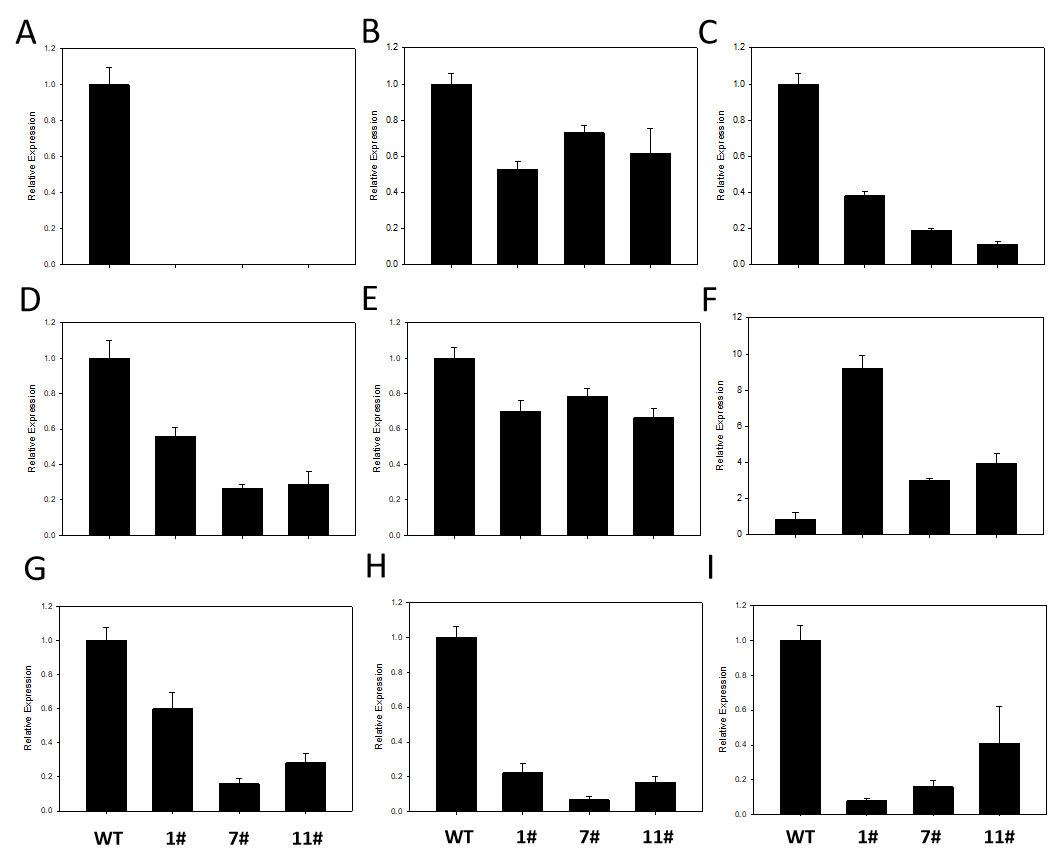

Supplement: Supplementary file 1 [file plants-13-00492-s001.zip › Figure S1.tif]

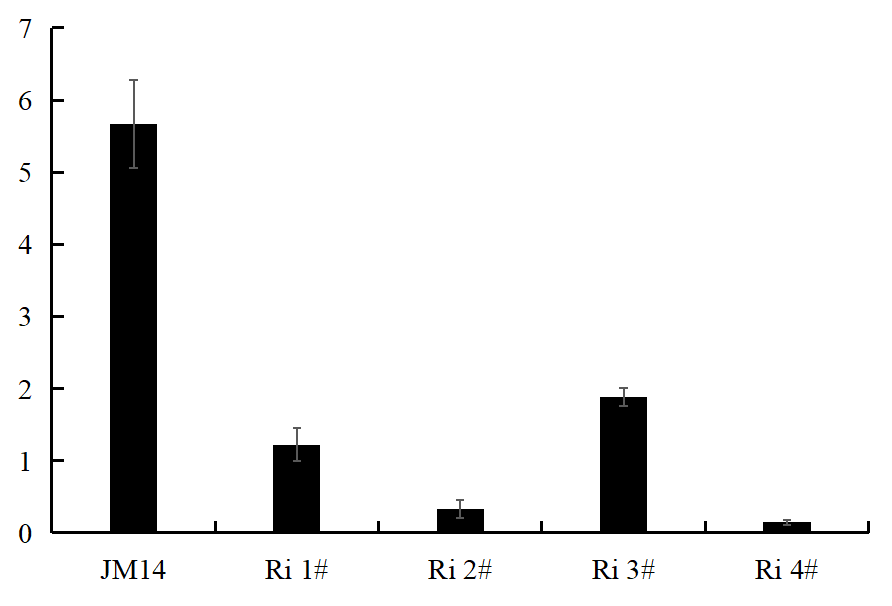

Supplement: Supplementary file 1 [file plants-13-00492-s001.zip › Figure S2.tif]
